# Effects of 5-Amyno-4-(1,3-benzothyazol-2-yn)-1-(3-methoxyphenyl)-1,2-dihydro-3H-pyrrol-3-one Intake on Digestive System in a Rat Model of Colon Cancer

**DOI:** 10.1155/2015/376576

**Published:** 2015-10-04

**Authors:** Halyna M. Kuznietsova, Valentyna K. Luzhenetska, Iryna P. Kotlyar, Volodymyr K. Rybalchenko

**Affiliations:** Institute of Biology, Taras Shevchenko National University, 64/13 Volodymyrska Street, Kyiv 01601, Ukraine

## Abstract

*Introduction*. Pyrrol derivate 5-amyno-4-(1,3-benzothyazol-2-yn)-1-(3-methoxyphenyl)-1,2-dihydro-3H-pyrrol-3-one (D1) has shown antiproliferative activities *in vitro*, so investigation of the impact of D1 intake on gut organs in rats that experienced colon cancer seems to be necessary. *Materials and Methods*. D1 at the dose of 2.3 mg/kg was administered per os daily for 27 (from the 1st day of experiment) or 7 (from the 21st week of experiment) weeks to rats that experienced 1,2-dimethylhydrazine (DMH)-induced colon cancer for 20 weeks. 5-Fluorouracil (5FU) was chosen as reference drug and was administered intraperitoneally weekly for 7 weeks (from the 21st week of experiment) at the dose of 45 mg/kg. *Results*. Antitumor activity of D1 comparable with the 5FU one against DMH-induced colon cancer in rats was observed (decrease of tumor number and tumor total area up to 46%). D1 attenuated the inflammation of colon, gastric and jejunal mucosa, and the liver, caused by DMH, unlike 5FU, aggravating the latter. In addition, D1 partially normalized mucosa morphometric parameters suggesting its functional restore. *Conclusions*. D1 possesses, comparable with 5-fluorouracil antitumor efficacy, less damaging effects on the tissues beyond cancerous areas and contributes to partial morphological and functional gut organs recovery.

## 1. Introduction

Cancer is one of the major public health problems in the world, becoming the leading cause for deaths among general population [1, 2]. Chemotherapy is the most common method for neoplasm treatment. However, it has significant disadvantages such as high frequency and severity of side effects. This is the one of the main factors for choosing the proper chemotherapy and for treatment outcomes [3, 4].

Protein kinases inhibitors recently attract the attention as inhibitors of the proliferative activity having specific effects on malignant cells and low toxicity towards normal cells in the organism. However information about their systemic exposure is controversial [5], which requires detailed studies on each case. Particularly it concerns the small molecular inhibitors of protein kinases, which are promising therapeutics because of their moderate specificity (have a wide range of malignant “targets”), ease of use (oral administration), and lower cost compared with monoclonal antibodies [6].

An important criterion of anticancer drug development, in addition to its efficacy, is no negative impact on the systemic condition after prolonged use. Digestive organs are first exposed to exogenous substances including drugs, especially if administered per os. Exactly the digestive system occurs most side effects of anticancer therapy. Gut epithelium is hypersensitive to the cytostatic action because of high proliferative activity [7], whereas the liver detoxicates the xenobiotics and often is damaged by their metabolites [8].

**Figure 1:**
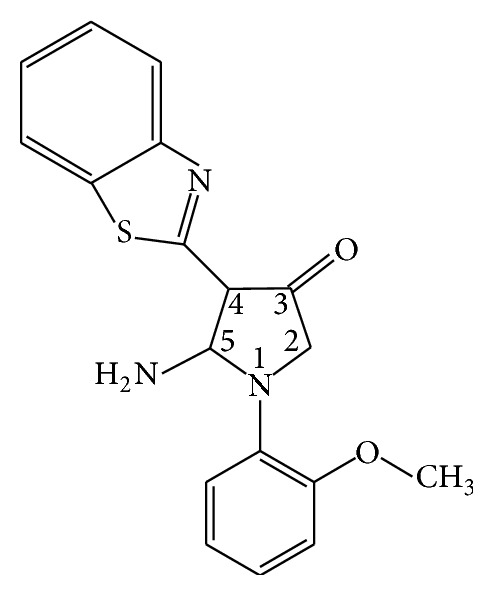
5-amyno-4-(1,3-benzothyazol-2-yl)-1-(3-methoxyphenyl)-1,2-dihydro-3H-pyrrol-3-one (D1) structural formula.

Pyrrol derivates have been synthesized at the Department of Chemistry of Taras Shevchenko National University of Kyiv as inhibitors of protein kinases such as Yes, Src(h), ZAP70, Syk(h), PDK1, EGFR, IGF-1R, and VEGFR [9, 10]. One of them, 5-amyno-4-(1,3-benzothyazol-2-yn)-1-(3-methoxyphenyl)-1,2-dihydro-3H-pyrrol-3-one, called D1 (Figure 1), has shown high cytostatic activity* in vitro* against HT29, HCT-15, and COLO-205 (colorectal cancer) cell lines, which suggests its potential anticancer activity. As D1 showed the most promising results on colorectal cancer cell lines, rat 1,2-dimethylhydrazine-induced colon cancer model was chosen for current* in vivo* investigations.

1,2-dimethylhydrazine/azoxymethane- (DMH/AOM-) induced colon carcinogenesis is a multistep process with morphological and histological features similar to those seen in human sporadic colon carcinogenesis [11, 12]. The main cellular and molecular defects found in human colon carcinogenesis have also been observed in DMH/AOM rat colon carcinogenesis. These alterations have been found to be involved in such pathways, as the Wnt pathway, K-ras pathway, TGF*β* signaling pathway, and inflammatory related process [13].

The Wnt pathway has been implicated as a crucial step in the initiation and development of colonic tumorigenesis. In the absence of the extracellular Wnt signal, free *β*-catenin is bound to the APC-axin-conductin-GSK3*β* complex. Phosphorylation of *β*-catenin by this complex marks it for ubiquitination and subsequent proteolytic degradation by the proteasome. When APC or *β*-catenin is mutated, *β*-catenin cannot be degraded but accumulates in the cytoplasm and translocates into the nucleus, where it binds to T-cell factor (TCF) and activates the Wnt target genes. Well-known downstream targets of APC/*β*-catenin/Tcf-4 mediated transcriptional activation in colorectal neoplasia include genes, such as cyclin D1, c-myc, which have important roles in proliferation, apoptosis, and cell cycle progression and are responsible for tumor formation.

Mutations in the* K-ras* gene are responsible for activation of the K-ras pathway. In human tumors, as well as in tumors of AOM-treated rats, increased expression of Akt, a downstream target of the K-ras pathway, has been found. PI3K/Akt pathway is implicated in glucose metabolism, cell proliferation, apoptosis, transcription, and cell migration. PI3K/Akt also promotes cyclin D1. In addition, activation of the MEK/MAPK/ERK pathway, another downstream K-ras signaling, has been found in tumors without K-ras mutation. Activation of this pathway has been associated with overexpression of c-erbB-2 receptors and decreased expression of GTPase activating protein, resulting in constitutive activation of normal K-ras protein. There are several bodies of evidence that Wnt/Apc/*β*-catenin/Tcf pathway is involved in COX-2 expression [14].

COX-2 expression in colorectal cancer is significantly higher than in normal colorectal tissues. COX-2 can (1) increase the production of prostaglandins and inhibit the body's immune response, (2) inhibit tumor cell apoptosis and promote cell proliferation, (3) regulate cell cycle progression, (4) promote tumor angiogenesis, (5) increase the expression of matrix metalloproteinases in tumor cells, and (6) induce activation of precursors of carcinogenic substances. AOM augments the expression of cyclooxygenase- (COX-) 2 and consequently prostaglandin E2 (PGE2) levels within adenocarcinomas and upregulates a number of proinflammatory cytokines, including tumor necrosis factor- (TNF-) a and interleukin-1a/b. Elevated COX-2 expression has been shown to occur throughout the carcinogenic process, beginning in rats within normal-appearing colonic mucosa as early as 1 week following AOM exposure [15]. COX-2 activates intrinsic tyrosine kinase of EGFR. EGF can form a complex with *β*-catenin, possibly through receptor tyrosine kinase-PI3K/Akt pathway, and further activate Wnt signaling pathway. Coss-communication between Wnt- and EGFR-signaling pathways allows the integration of the diversity of stimuli in colonocytes and promotes tumor progression [16]. TGF*β* in normal cells stops the cell cycle at the G1 stage, induces differentiation or promotes apoptosis, and thus exerts tumor suppressive effects. In cancer cells, it also modulates processes such as cell invasion, immune regulation, and microenvironment modification that cancer cells may exploit to their advantage. It has been found that TGF*β* and COX-2 were concurrently overexpressed in the same colonic neoplastic lesions in AOM-treated rats, suggesting that COX-2 expression in AOM-induced colonic tumor could, in part, be due to the overexpression of TGF*β* [13].

Colon is the tissue where carcinogenesis processes pass, so we suggested, that conditions in the colon differ from that ones in other parts of digestive system. Therefore the outcome of the latter under colon cancer conditions has to be studied. So investigation of the impact of pyrrol derivate D1 intake on the colon, gastric and jejunal mucosa, and the liver in rats that experienced colon cancer was aimed. 5-Fluorouracil commonly used for colorectal cancer treatment [7, 17] was chosen as reference drug.

## 2. Materials and Methods

### 2.1. Animals

All experimental procedures executed with animals were in compliance with international principles of the European Convention for the protection of vertebrate animals used for experimental and other scientific purposes (European convention, Strasburg, 1986), article 26 of the Law of Ukraine “on protection of animals from cruelty” (number 3447-IV, 21.02.2006), and all norms of bioethics and biosafety.

80 Male Wistar rats weighing 120–130 g (4 weeks old) were obtained from Central Animal House of Taras Shevchenko National University (Kyiv, Ukraine), and 5 animals were housed per plastic cage on softwood chip bedding. These animals were maintained under constant conditions (12 hr light/dark cycle, 60% humidity at 20–22°C) and fed on standard diet and tap water* ad libitum*.

### 2.2. Chemicals

D1 was dissolved in vegetable oil containing 15% dimethylsulfoxide. Animals were treated by D1 at the dose of 2.3 mg/kg of body weight* per os* daily. The common anticancer therapeutic 5-fluorouracil (5FU, Ebewe Pharma, Austria) was used for referencing. Undiluted 5FU was injected intraperitoneally weekly at the dose of 45 mg/kg of body weight [18]. 1,2-Dimethylhydrazine (DMH, “Acros Organics”, USA), a highly specific colorectal carcinogen in rodents, was dissolved immediately before use in saline adjusted to pH 6.5 with sodium hydroxide. To induce tumor development, animals were subcutaneously injected with 20 mg/kg DMH weekly for 20 weeks [19].

### 2.3. Experimental Design

Colorectal tumors were induced as described [19]: animals were administered with DMH subcutaneously during the first 20 weeks of experiment; then tumor development followed for 7 weeks. D1 applications were started simultaneously with the administration of DMH and followed for 27 weeks or at the 21st week of experiment and followed for 7 weeks. 27-week intake was called as preventive regimen and started from the 1st day of experiment, and 7-week intake was called as therapeutic regimen and started from the 21st week of experiment, when the first tumor nodes appear. 5FU injections were started at the 21st week of experiment and followed for 7 weeks. Control animals received the appropriate vehicles: saline or vegetable oil that contained 15% dimethylsulfoxide.

The rats were divided into 8 groups (10 rats each): (1) vehicle-treated control, (2) D1^7  weeks^, where the animals were treated with D1 for 7 weeks starting at the 21st week of experiment, (3) D1^27  weeks^, where the animals were treated with D1 for 27 weeks, (4) 5FU, where the animals were treated with 5FU for 7 weeks starting at the 21st week of experiment, (5) DMH, where the animals were treated with DMH for 20 weeks and then tumor development followed for 7 weeks, (6) DMH + D1^7  weeks^, where the animals were treated with DMH for 20 weeks and with D1 for 7 weeks starting at the 21st week, (7) DMH + D1^27  weeks^, where the animals were treated with DMH for 20 weeks and with D1 for 27 weeks starting at the 1st day of experiment, and (8) DMH + 5FU, where the animals were treated with DMH for 20 weeks and with 5FU for 7 weeks starting at the 21st week of experiment.

### 2.4. Tissue Preparation

One day after the last treatment the rats were sacrificed by carbon dioxide asphyxia, the abdomen was opened, and the entire gastrointestinal tract and liver were removed. The bowel internal side was examined, the tumor number per animal and the areas of tumors were measured, and the tumor total area per animal was calculated.

The colon segments with no tumors, the jejunum ones, and the fundus parts of the stomach were fixed for 14 days in neutral saline containing 10% formalin; the liver samples were fixed for the same time in Bouin's fixative. Then, they were embedded into paraffin and sliced into 5 *μ*m sections, which were stained with hematoxylin-eosin-orange [20] and examined under the light microscope (Olympus BX-41, Olympus Europe GmbH, Japan). Systemic conditions of colon, gastric and jejunal mucosa, and the liver were assessed. Gastric mucosa thickness, cross-sectional areas of chief and parietal cells and their nuclei, colon and jejunal mucosa thickness, colonocytes/enterocytes height and their nuclei cross-sectional area, as well as goblet cells one, and cross-sectional areas of hepatocytes and their nuclei from centrolobular and periportal hepatic zones separately and hepatic sinusoid's diameter were measured from the microphotographs with magnification 400x, using WCIF ImageJ software. The mitotic index (MI) was calculated as the sum of epithelial cells in any phase of mitosis divided by the total sum of epithelial cells. No less than 300 colon or jejunal crypt epithelial cells per animal were assessed.

### 2.5. Liver Function Tests

Immediately after animals sacrifice the blood from inguinal vein was collected, kept for 20 min to coagulate, and then centrifuged for 5 min at 1000 g. Blood serum alanine aminotransferase (ALT), aspartate aminotransferase (AST), alkaline phosphatase (ALP), and lactate dehydrohenase (LDH) were determined by standard kits (“Reagent”, Ukraine). De Ritis coefficient was defined as AST/ALT ratio.

### 2.6. Urinary 8-oxoG Extraction Test

Urinary 7,8-dihydro-8-oxoguanine (8-oxoG) is one of the main products of DNA oxidative damage and an indicator of tumor availability and progression as well as drugs genotoxicity [21, 22]. Urinary 8-oxoG was determined as described [21]: animals were kept for 1 day in the urine collection chamber (on a standard diet with free access to water), collected urine was filtered, and daily volume was measured; 8-oxoG was extracted from urine aliquots (1 mL) by its filtration through a cartridge filled with a DSC-18 (Cayman Chemical Company, USA) followed by washing with methanol; 8-oxoG amount was determined spectrophotometrically; daily urine 8-oxoG extraction per 1 kg of body weight was calculated.

### 2.7. Statistical Analysis

Statistical analysis was carried out using SPSS 17.0 software for Windows. One-way ANOVA followed by the* post hoc* Bonferroni's test was employed to determine statistical significance. *p* < 0.05 was considered statistically significant.

## 3. Results

### 3.1. D1 Influence on the Digestive Organs of Healthy Rats

#### 3.1.1. Colon

Submucosa and lamina propria's slight inflammation manifested by lymphatic and histiocytic cells infiltration loci, as well as no epithelium damage, caused by D1 acting for 7 and 27 weeks, were observed. D1 acting for 7 weeks increased mucosa thickness, colonocyte height, and their nuclei area by 11–19%, whereas D1 acting for 27 weeks decreased mucosa thickness by 12% and MI by 27%, suggesting the depletion of adaptive reserves and inhibition of cell proliferation. On the contrary, moderate lymphatic and histiocytic cells infiltration of the upper mucosa layer and sometimes blood capillary dilation were observed in 5FU group, suggesting the inflammatory process. 5FU increased colonocyte height (by 11%) as D1^7  weeks^ did but also inhibited cell proliferation, manifested by MI decrease by 37% (Table 2).

#### 3.1.2. Stomach

Moderate submucosal and lamina propria's edema, isolated inflammation features manifested by lymphatic and histiocytic cells infiltration loci, caused by D1 acting for 7 and 27 weeks, were observed. Some areas of surface epithelium desquamation also appeared. On the contrary, 5FU caused severe mucosa damage, manifested by surface epithelium desquamation, gastric glands' destruction, sometimes colonocytes dystrophy, and necrosis foci. D1 increased the values almost of all measured parameters in time-dependent manner by 6–13% acting for 7 weeks and by 11–22% acting for 27 weeks (Table 3), which could indicate mucosa compensatory processes by intensification of synthetic processes in colonocytes [23]. However decrease of all measured parameters by 11–17% together with morphological changes, caused by 5FU, could indicate chronic atrophic gastritis development in 5FU group.

#### 3.1.3. Jejunum

There were slight edema, hyperaemia, and sometimes stroma lymphatic and histiocytic cells infiltration in jejunal villi. In D1^7  weeks^ group no changes were observed compared to control one, whereas in D1^27  weeks^ group goblet cells' and enterocytes nuclei areas were reduced by 15% and 24%, respectively, supposing mucosa functional activity inhibition (Table 4). On the contrary, 5FU caused edema of villi apexes, epithelium desquamation, inflammation, enterocytes nuclei area reduction by 12.7% together with their height increase by 13.4%, and intensification of progenitor cells proliferation by 35.4%, which evidenced chronic enteritis development.

#### 3.1.4. Liver

D1 acting for 7 weeks sometimes caused sinusoids inflammatory dilation, in some cases granular dystrophy. Hepatocytes area increase by 12% and 13%, respectively, has been shown in both hepatic zones (Table 3), indicating the activation of detoxificating processes [24]. In D1^27  weeks^ group no inflammation and dystrophy features were observed, although centrolobular and periportal hepatocytes areas were increased by 10% and 9%, respectively, indicating partial liver adaptation and its structure recovery while continuing detoxificating processes. Cell cytoplasm inhomogeneity especially in periportal zone, hyperchromic nuclei, and lymph infiltration of perivascular areas caused by 5FU could evidence the drug hepatotoxicity. Hepatocytes area increase by 12% has been shown in periportal zone that indicated the liver detoxificating activity (Table 5). D1 decreased serum ALT by 20–22%, when administered for both terms, confirming the morphological data about liver functional load associated with the process of xenobiotics detoxification [25]. Statistically significant changes of serums ALP, AST, and LDH were not observed, confirming no D1 pronounced hepatotoxicity. On the contrary, 5FU decreased serum AST by 23% and did not affect other measured enzymes (Table 6). Furthermore, in contrast to classical anticancer drugs [26] having strong genotoxicity, D1 did not induce significant DNA oxidative damage, evidenced by a moderate (by 33–46%) urinary 8-oxoG increase. 5FU increased urinary 8-oxoG up to 5 times towards 0.49 ± 0.07 nmol/kg body weight per day in control.

### 3.2. Antitumor Action of D1

Visual inspection of the bowels of rats that experienced colon cancer detected tumors mainly in the distal bowel part and predominantly of exophytic type, which is consistent with published data [19]. All tested compounds, D1^7  weeks^, D1^27  weeks^, and 5FU, reduced the tumor total area at a similar manner (by 40–46%) (Table 1). Statistically significant decrease of tumor number was found in DMH + D1^7  weeks^ and DMH + 5FU groups (by 41 and 50%, resp.). Moreover, predominantly exophytic tumors were affected by tested compounds. Thus, antitumor effects of D1 and 5FU were similar. Notably, antitumor effect of D1 acting for 7 weeks was stronger comparing to 27-week treatment, which may be explained by adaptation to D1 under its prolonged influence.

### 3.3. D1 Influence on the Digestive Organs of Rats That Experienced Colon Cancer

#### 3.3.1. Colon

**Figure 2:**
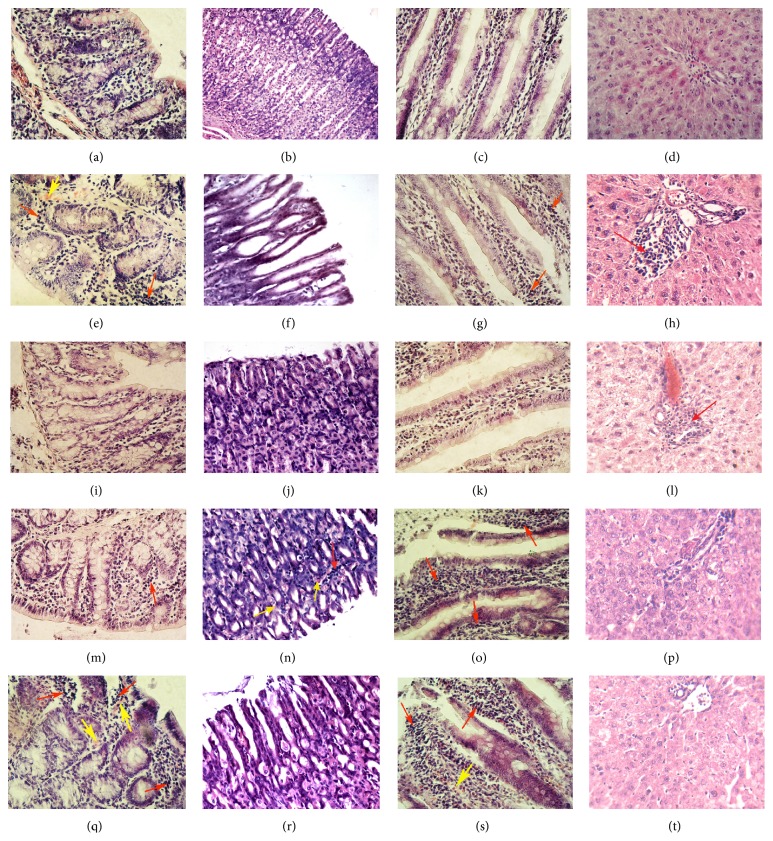
Microphotographs of rat colon ((a), (e), (i), (m), and (q)), gastric ((b), (f), (j), (n), and (r)) and jejunal ((c), (g), (k), (o), and (s)) mucosa, and liver periportal zone ((d), (h), (l), (p), and (t)); hematoxylin-eosin-orange stain; magnification 400x. Experimental groups: control: (a), (b), (c), and (d); DMH: (e), (f), (g), and (h); DMH + D1^7  weeks^: (i), (j), (k), and (l); DMH + D1^27  weeks^: (m), (n), (o), and (p); DMH + 5FU: (q), (r), (s), and (t). Inflammatory features in groups DMH ((e), (g), and (h)), DMH + D1^7  weeks^ (l), DMH + D1^27  weeks^ ((m), (n), and (o)), and DMH + 5FU ((q) and (s)) are manifested by lymphatic and histiocytic cells infiltration, marked by red arrows, and blood capillary dilations, marked by yellow arrows. Damage of surface epithelium appeared in groups DMH ((f) and (g)) and DMH + 5FU ((q), (r), and (s)) and appeared less in DMH + D1^27  weeks^ (o).

Inflammatory features, manifested by lymphatic and histiocytic cells infiltration and blood capillary dilation (Figure 2(e)), increased mucosa thickness, colonocyte height, and their nuclei area (by 13–25%); sometimes enlarged and overextended crypts in DMH group could be interpreted [27] as precursor lesions. Local lymphatic and histiocytic cells aggregations in DMH + D1^27  weeks^ group (Figure 2(m)) and no changes in DMH + D1^7  weeks^ group (Figure 2(i)) were observed comparing to control. On the contrary, inflammation was enhanced in DMH + 5FU group (Figure 2(q)): the lymphatic and histiocytic cells infiltration was more prevalent and the vessel dilations were more frequent. Damage of surface epithelium was also observed in this group. D1^7  weeks^ increased colonocyte height and their nuclei area (by 14% and 6%, resp.), suggesting the cells functional activity gain, and didn't affect the cell proliferation. Otherwise, D1^27  weeks^ and 5FU increased all mucosa parameters by 10–24%, except for MI, which was diminished by 29% and 32%, respectively (Table 2).

#### 3.3.2. Stomach

Severe epithelium desquamation and lymphatic and histiocytic cells submucosal infiltration were observed in DMH-treated rats. Reduce the number of parietal cells due to relatively deep mucosal defects (Figure 2(f)) and decrease the values of all parameters by 19–39% (Table 3) were also detected. These findings suppose [23] chronic atrophic gastritis and therefore mucosa functional activity suppression.

In D1-treated rats that experienced colon cancer some submucosal edema and lymphocytes and histiocytes aggregations were discovered. Epithelium desquamation was observed on minor areas in D1^7  weeks^ group and on almost the entire surface in D1^27  weeks^ group (Figures 2(j) and 2(n)). On the contrary, 5FU caused severe epithelium desquamation, cell dystrophy and necrosis foci, and focal lymphatic and histiocytic cells submucosal infiltration (Figure 2(r)).

D1 acting for 7 weeks caused an increase of all tested parameters by 6–30% compared to DMH, which became close to control values but remained below them by 6–28%. D1 acting for 27 weeks also caused an increase of all tested parameters by 6–20% remaining lower than control one by 14–35%. In DMH + 5FU group similar effects were observed: mucosa parameters were increased by 8–40% remaining lower than control ones by 9–18% (Table 3). Recovery effects of D1 acting for 7 weeks were more pronounced regarding to chief cells, in comparison with 5FU, as well as with D1 more prolonged action. However, parietal cells were more sensitive to D1 acting for both terms compared to 5FU.

So D1 partially recovers gastric mucosa altered by DMH, unlike 5FU, which aggravates carcinogenesis consequences in the stomach.

#### 3.3.3. Jejunum

The features of impaired capillary blood and lymph circulation and inflammation accompanied with villi apexes edema were observed in rats that experienced colon cancer (Figure 2(g)). Enterocytes nuclei area was reduced by 13% (Table 4) and mucosal cell proliferation wasn't changed in this group. These findings indicate [23] the development of chronic enteritis without atrophy.

D1 acting for both terms did not affect the violation of mucosal lymphatic drainage but contributed to villi apexes edema reduction, when administered for 7 weeks (Figures 2(k) and 2(o)). Enterocyte height increase by 10–13% was accompanied with their nuclei area and goblet cells area decrease by 15% and 11%, respectively (Table 4), and therefore could indicate further mucosa functional activity inhibition in comparison with untreated animals.

Unlike D1, 5FU aggravated mucosa blood and lymphatic capillary disturbances and inflammatory features (Figure 2(s)). Decrease of cell morphometric parameters by 8–14% towards DMH and by 13–26% towards control, caused by 5FU, as well as inhibition of cell proliferation by 30% towards DMH, was observed. This could indicate the inhibition of mucosa functional activity and regeneration. So both of tested cytostatics inhibit jejunal mucosa functional activity. Nevertheless, compensatory processes, manifested by increase of mucosa thickness, enterocytes height, and mitotic index by 10–25%, occurred in D1 groups (Table 4).

#### 3.3.4. Liver

Substantial liver violations, caused by DMH, appeared: liver histoarchitectonics disrupture, sometimes hepatocytes hydropic dystrophy in periportal zone, venous congestion, and significant lymphocytes and histiocytes infiltration of the portal tracts (Figure 2(h)) could be the features of chronic hepatitis [23]. Serum ALP and ALT increase by 50% and 20%, respectively, accompanied with AST decrease by 23% (Table 6), indicated liver lesions [8, 23]. Hepatocytes and their nuclei areas from both zones were increased by 13–23% (Table 5), supposing detoxification arise.

Histological analysis showed the dominance of DMH impact in D1^7  weeks^ group: liver girder structure was destroyed, inflammatory features were observed in the portal tracts, hemocapillars were expanded, and hepatocyte cytoplasm was inhomogeneous (Figure 2(l)). The consequences of DMH impact were also stored in D1^27  weeks^ group as venous and sometimes sinusoids congestion and inhomogeneous hepatocyte cytoplasm. Nevertheless, lymphocytes and histiocytes infiltration of perivascular areas was much less pronounced (Figure 2(p)). In 5FU group injuries of liver girder structure and inflammatory features persisted; also liver granular degeneration and hepatocytes edema occurred (Figure 2(t)), indicating the aggravation of DMH-induced liver tissue injuries.

Hepatocytes and their nuclei area in D1 and 5FU groups were increased by 15–26% and 12–24%, respectively, towards control ones, indicating the intensity of detoxification processes. D1 acting for both terms did not affect serums ALT and LDH but contributed ALP and AST close to control ones. The effects of 5FU on these enzymes were the same. Hereby the effects of both investigated cytostatics on liver tissue were similar: although the carcinogen-induced effects dominated on the structural level, enzyme activities changes indicated the partial recovery of liver function.

In DMH group significant (up to 5-fold) urinary 8-oxoG increase was determined, which is consistent with the literature [26]. D1, when administered to rats that experienced colon cancer, partially prevented DNA oxidative damage, reducing urinary 8-oxoG by 18–21% (*p* = 0,035 for D1^7  weeks^ and *p* = 0,029 for D1^27  weeks^), which could indicate the antioxidant properties of the chemical. On the contrary, 5FU did not affect the elevated urinary 8-oxoG.

## 4. Discussion

Histological analysis evidences D1 minor toxicity to mucosa of upper gastrointestinal tract following subchronic exposure. However D1 effects were compounded following chronic one, obviously because of compensatory-adaptive reserves depletion [17]. At the same time, D1 chronic exposure was less toxic to the liver, presumably because of its adaptation and recovery. TKIs are primarily metabolized in the liver by CYP enzymes (mainly CYP3A4) and are eliminated via the biliary excretion route in the feces as unchanged drug or metabolites by the agency of ATP-binding cassette (ABC) transporters, such as the breast cancer resistance protein (BCRP) or P-glycoprotein [25]. Hepatic uptake of drugs is mediated via membrane transporters, localized on the basolateral side of hepatic tissue. This uptake process is recognized as the first step in hepatocellular elimination and plays a vital role in hepatic drug disposition. Organic anion transporting polypeptides (OATPs) appear to play a critical role in bioavailability, distribution, and excretion of numerous exogenous amphipathic organic anionic compounds including many drugs. OATPs, namely, OATP-1B1 and OATP-1B3, are responsible for uptake of TKIs into human liver cells. However, some of the TKIs, namely, pazopanib and lapatinib, are known to inhibit the functional capacity of OATP-1B1 and/or OATP-1B3 transporter proteins [28]. However, some TKIs could inhibit CYP3A4, CYP2C8, CYP2C9, CYP2D6, and uridine diphosphate glucuronosyltransferase 1A1 (UGT1A1), potentially increasing the concentrations of drugs eliminated by these enzymes [29, 30]. Moreover, some small-molecule TKIs have been found to inhibit ABC transporters [25]. Such capability, of course, could be used to overcome anticancer drug resistance, and these data were obtained in recent years [31]. But in our case we may suggest that probably inhibition of ABC transporter by D1 together with different reactions of CYP cytochromes and OATPs could contribute to D1 accumulation in the liver and thus to some hepatotoxicity of the chemical.

DMH carcinogenic properties are caused by capability of its reactive metabolite, methyldiazonium ion, to methylate nucleic acids, histones, and other DNA-binding proteins. [19]. Liver reasonably high damage degree under DMH-induced carcinogenesis occurs because of DMH metabolic activation to methyldiazonium ion in this organ [19]. Methyldiazonium ion is an inducer of free radical oxidation. Accordingly, liver the first undergoes to its destructive influence. In addition, tumor growth increases liver functional load by releasing toxic products of malignant cells. D1 did not protect rat liver from DMH-induced injury: hepatitis and significant liver functional load features persisted, although the inflammation intensity was reduced. The persistence of liver hepatotoxicity could be explained by intensive xenobiotic detoxification (as D1 as DMH). D1 could also modify the activities of P450 cytochromes and transporters, which are responsible for DMH input and output, and therefore contribute to liver hepatotoxicity.

Some studies have demonstrated that DMH not only causes DNA mutations, predominantly due to O^6^MeG formation, providing cell malignant transformation or apoptosis, but also inhibits repair enzymes including O(6)-alkylguanine-DNA alkyltransferase, which is expressed variously in different tissues [32]. Hereby we could suggest that atrophic features development in the gastric mucosa could be determined by strengthened apoptosis due to impaired DNA repair, while the jejunal mucosa avoids the atrophy through more intensive DNA repair.

In our study the rats from the DMH-induced cancer groups exhibited tumor localization mainly in the distal colon, and the observed tumors were mainly exophytic, which is in good agreement with [27, 33]. Exophytic tumors were predominantly localized in distal colon, when endophytic ones were localized in proximal colon. It was found that D1 and 5FU mainly affect the growth of exophytic tumors (adenomas and hyperplastic polyps).

It has been found that proximal colonic neoplasms in humans have distinct characteristics in terms of histology and molecular genetics. They are generally less differentiated and have a higher propensity for microsatellite instability than distal ones. Histologically, tumors in the distal colon are mostly adenomas and well or moderately differentiated adenocarcinomas; in the proximal colon, tumors are mostly poorly differentiated, mucinous, or signet-ring cell adenocarcinomas. It is believed that, in the middle and distal colon, histogenesis follows aberrant crypt foci-adenoma-carcinoma sequences, while in the proximal colon, poorly differentiated mucin-secreting carcinomas arise de novo without an intermediate stage of colon carcinogenesis. Furthermore, proximal and distal tumors in the DMH/AOM rat model also have distinct characteristics in terms of kinetic features, sensitivity to carcinogens, and chemotherapy. Thus, COX-2 expression demonstrated a 3- to 4-fold excess in the distal relative to the proximal bowel in DMH/AOM-treated rats, as well as peroxisome proliferator-activated receptor-delta (PPAR-delta) [34]. So we propose that different sensitivity of adenomas/adenocarcinomas and carcinomas is caused by differences in COX-2 expression.

It was suggested that intestinal epithelial cells upregulated COX-2 expression in a TLR4- and MyD88-dependent fashion. This signaling is required for optimal proliferation and protection against apoptosis in the injured intestine, while TLR4 may lower the threshold for carcinogenesis in an AOM-induced cancer model [35]. So we could suggest more expression and/or activation of TLR-4/MyD88 in adenomas and adenocarcinomas. As many of protein kinases are involved in TLR-4/MyD88-dependent pathway (IRAK1, IRAK4, TBK1, and IKKi), which leads to overexpression of COX-2, we suggest that D1 could inhibit COX-2 expression through inhibition of these kinases. The mechanism of 5FU action could be different: 5FU could inhibit COX-2 expression through inhibition of nucleotide synthesis. It was found that the expression of COX-2 was significantly decreased following treatment with 5FU monotherapy [36], although little amount of proximal tumors also contributes to statistical insignificance of the results.

D1 reduces the tumor number and total tumor lesions area at 27th week by preventing new tumor formation and by regress of existing ones. The effects of 5FU are similar [37].

D1 antitumor activity could be realized through inhibition of COX-2 expression, as mentioned above. Moreover, D1 is hydrophobic compound and so could integrate and accumulate in the lipid phase of cellular membrane and interact with membrane-associated protein kinases, such as EGFR, VEGFR, and IGF1R (insulin-like growth factor-1 receptor), which are overexpressed or hyperactivated in colonic tumor cells. Thus, DMH causes a 12-fold increase in EGFR expression in the colonic mucosa, when compared with the corresponding controls. EGFR signaling is coupled directly or via adaptor proteins to MAPK signaling, PI3/AKT pathway, which plays a crucial role in DMH/AOM-induced colon carcinogenesis, JAK/STAT (Janus kinase/signal transducer and activator of transcription) pathways. EGFR signaling also modulates expression of VEGF [38]. VEGFR is the predominant proangiogenic cytokine in colon cancer. VEGF stimulation results in enhanced cellular migration, but this could be blocked by inhibition of VEGFR. IGF1R activation leads to activation of signaling cascades including the GRB2/Ras/ERK and IRS-1/PI3K/AKT pathways. Of note, IGF-2 is overexpressed in many solid tumors including colon cancer [16]. D1 could also inhibit PDK1, which is the first node of the PI3K signal output and is required for activation of AKT. Increased PDK1 potentiates AKT signaling in the setting of upstream PI3K pathway activation. PDK1 levels had their most prominent potentiating effect on the PI3K signal due to an upstream pathway lesion when growth factor input was low. PDK1 overexpression in tumors increases the level of oncogenic PI3K signal due to pathogenetic activation of PI3K [39].

The next potential target of D1 inhibition is Src. Src expression is increased in approximately 80% of colorectal cancer specimens compared with normal colonic epithelium. Src activity in primary colon carcinomas was 5- to 7-fold higher than normal colonic mucosa adjacent to the tumor. The multiple effectors of Src include the PI3K/Akt, Ras/Raf/MAPK, STAT3/STAT5B, and p130 pathways; moreover, VEGFR promotes migration of tumor cells through a Src-dependent pathway [40].

D1 treatment partially neutralized the effects of carcinogen impact, reducing inflammatory features in the colon, gastric, and jejunal mucosa, whereas 5FU escalated this process. Anti-inflammatory action of D1 could be realized through its protein kinase inhibitory properties. Thus, EGFR, which is a target of D1, acts as the major upstream activator of phosphatidylinositol 3-kinase (PI3K)/Akt pathway leading to activation of NF-*κ*B, which has an essential role in inflammation and innate immunity [41]. Anti-inflammatory effect of D1 also was discovered in our previous studies [42].

On the other hand, 5FU escalates DMH-induced inflammation in gut mucosa. There are several bodies of evidence, that 5FU significantly activated the NF-*κ*B activity in the small intestine [43]. But other authors found that 5FU suppresses NF-*κ*B by mediating the upregulation of I*κ*B-*α* expression [44]. Nevertheless, elevation of TNF-*α* and IL-1*β*, proinflammatory cytokines, was shown in mucosal tissue in 5FU treated rats [45]. So inflammatory changes in 5FU-induced mucositis may be also determined by pathways independent of NF-*κ*B.

As D1, as 5FU realizes their toxicity by apoptosis induction, the mechanisms of such action could be different. 5FU acts in several ways but principally as a thymidylate synthase inhibitor. Interrupting the action of this enzyme blocks synthesis of the pyrimidine thymidine, which is a nucleoside required for DNA replication. Administration of 5FU causes a scarcity in deoxythymidine monophosphate (dTMP), so rapidly dividing cancerous cells undergo cell death via thymineless death. Active metabolite of 5FU fluorodeoxyuridine triphosphate (FdUTP) also could incorporate into DNA via DNA synthesis and cause DNA damage and cell cycle arrest through mismatch repair mechanism. And thus apoptosis of such cells occurs [46]. 5FU could also induce ROS and ROS-dependent Src activation in colon cancer cells. Of course, Src has been reported to inhibit apoptosis by activating the PI3k/Akt pathway, which protects cells against proapoptotic stimuli through the phosphorylation and inactivation of death accelerators, such as Bad, Bax, and caspase-9. On the other hand, Src has also been reported to enhance apoptosis induced by different proapoptotic stimuli, and this effect is triggered specifically at high Src signaling levels [47]. Moreover, 5FU-induced intestinal damage initiates a proinflammatory process and it is likely that inflammatory cytokines mediate the subsequent apoptosis [45].

D1-induced apoptosis is a result of inhibiting protein kinases, involved in pathways, responsible for cell survival (RAS/Raf/MEK/ERK, PI3K/AKT), which are described above. The proapoptotic properties of D1 against epithelial cells were demonstrated in [48].

## 5. Conclusions

No expressed toxicity of pyrrol derivate 5-amyno-4-(1,3-benzothyazol-2-yn)-1-(3-methoxyphenyl)-1,2-dihydro-3H-pyrrol-3-one (D1) to the gut organs when administered for 7 or 27 weeks was shown, unlike the reference therapeutic 5-fluorouracil (5FU). Antitumor effects of D1 and 5FU were similar, indicating the effectiveness of D1 as antineoplastic agent. D1, when administered to rats experienced DMH-induced colon cancer, attenuates the inflammation of gastric, jejunal, and colon mucosa and hereby protects these tissues against DMH-induced injury. On the other hand, 5FU, administered by the same manner, aggravates the inflammatory features. In addition, D1 partially normalizes gastric, colon, and jejunal mucosa morphometric parameters suggesting the mucosa functional restore.

Thus, D1 possesses, comparable with 5-fluorouracil antitumor efficacy, less damaging effects on the tissues beyond cancerous areas, contributes to partial morphological and functional gut organs recovery, and so could be recommended for further investigation as active ingredient of new anticancer drugs.

## Figures and Tables

**Table 1:** Tumor parameters for rats treated by D1 and 5FU under DMH-induced carcinogenesis. Mean ± SEM, *n* = 10^#^.

	DMH	DMH + D1^7 weeks^	DMH + D1^27 weeks^	DMH + 5FU
Total tumors				
Tumor number	9.56 ± 1.74	5.60 ± 1.27 (*p* = 0.015)	7.17 ± 1.70	4.78 ± 1.44 (*p* = 0.003)
Tumor total area, cm^2^	1.58 ± 0.65	0.94 ± 0.40 (*p* = 0.025)	0.86 ± 0.47 (*p* = 0.019)	0.91 ± 0.68 (*p* = 0.038)

Exophytic tumors				
Tumor number	9.00 ± 1.53	4.90 ± 1.38 (*p* = 0.004)	6.50 ± 2.23	4.44 ± 1.42 (*p* = 0.001)
Tumor total area, cm^2^	1.03 ± 0.28	0.49 ± 0.16 (*p* = 0.016)	0.56 ± 0.37 (*p* = 0.023)	0.46 ± 0.21 (*p* = 0.013)

Endophytic tumors				
Tumor number	0.67 ± 0.47	0.70 ± 0.60	0.67 ± 0.67	0.33 ± 0.31
Tumor total area, cm^2^	0.55 ± 0.53	0.45 ± 0.42	0.3 ± 0.27	0.45 ± 0.42

^#^ *p* values indicated in comparison with DMH group.

**Table 2:** Morphometric parameters of colon mucosa, adjacent to colonic tumors for rats treated by D1 and 5FU under DMH-induced carcinogenesis. Mean ± SEM, *n* = 10^#^.

	Mucosa thickness, *μ*m	Colonocytes height, *μ*m	Colonocytes nuclei cross-sectional area, *μ*m^2^	Goblet cells cross-sectional area, *μ*m^2^	Mitotic index, %
Control	210.64 ± 9.62	16.75 ± 0.87	14.93 ± 1.22	107.08 ± 11.3	4.25 ± 0.32

D1^7 weeks^	233.12 ± 10.39 (*p* = 0.01)	18.69 ± 0.70 (*p* = 0.02)	17.83 ± 1.31 (*p* = 0.002)	99.01 ± 8.73	3.58 ± 0.32

D1^27 weeks^	186.00 ± 7.9 (*p* = 0.006)	17.04 ± 0.63	15.16 ± 0.84	99.76 ± 7.95	3.10 ± 0.43 (*p* = 0.047)

5FU	208.61 ± 9.18	18.53 ± 0.92 (*p* = 0.01)	14.09 ± 0.61	99.67 ± 7.59	2.66 ± 0.75 (*p* = 0.01)

DMH	238.56 ± 11.64 (*p* = 0.005)	20.93 ± 1.18 (*p* = 0.001)	18.59 ± 1.03 (*p* = 0.001)	108.01 ± 9.27	4.20 ± 0.26

DMH + D1^7 weeks^	209.48 ± 9.35 (^*∗*^ *p* = 0.007, ^$^ *p* = 0.001)	19.05 ± 0.84 (*p* = 0.004)	15.80 ± 0.81 (^*∗*^ *p* = 0.01, ^$^ *p* = 0.044)	99.96 ± 6.72	3.70 ± 0.35 (^$^ *p* = 0.041)

DMH + D1^27 weeks^	257.97 ± 11.64 (*p* = 0.001, ^*∗*^ *p* = 0.002, ^$^ *p* = 0.044)	18.60 ± 0.88 (*p* = 0.03, ^*∗*^ *p* = 0.04)	14.60 ± 1.08 (^*∗*^ *p* = 0.008, ^$^ *p* = 0.038)	130.63 ± 14.3 (*p* = 0.002, ^*∗*^ *p* = 0.002, ^$^ *p* = 0.001)	3.03 ± 0.24 (*p* = 0.03, ^*∗*^ *p* = 0.045)

DMH + 5FU	282.65 ± 12.95 (*p* = 0.001, ^*∗*^ *p* = 0.001)	18.47 ± 0.7 (*p* = 0.01, ^*∗*^ *p* = 0.009)	18.08 ± 1.11 (*p* = 0.005)	100.91 ± 6.78	2.87 ± 0.23 (*p* = 0.02, ^*∗*^ *p* = 0.021)

^#^ *p* values indicated in comparison with control; ^*∗*^compared to DMH group; ^$^compared to DMH + 5FU group: for DMH + D1^7  weeks^ and DMH + D1^27  weeks^ groups.

**Table 3:** Morphometric parameters of gastric mucosa for rats treated by D1 and 5FU under DMH-induced carcinogenesis. Mean ± SEM, *n* = 10^#^.

	Mucosa thickness, *μ*m	Chief cells cross-sectional area, *μ*m^2^	Chief cells nuclei cross-sectional area, *μ*m^2^	Parietal cells cross-sectional area, *μ*m^2^	Parietal cells nuclei cross-sectional area, *μ*m^2^
Control	512.77 ± 3.8	46.97 ± 1.4	10.03 ± 0.28	121.43 ± 2.32	17.03 ± 0.46

D1^7 weeks^	572.38 ± 2.34 (*p* = 0.002)	50.25 ± 1.36 (*p* = 0.035)	10.02 ± 0.27 (*p* = 1.000)	138.05 ± 2.32 (*p* = 0.003)	18.12 ± 0.34 (*p* = 0.039)

D1^27 weeks^	583.01 ± 1.84 (*p* = 0.001)	54.96 ± 0.74 (*p* = 0.004)	11.41 ± 0.3 (*p* = 0.041)	142.40 ± 3.61 (*p* = 0.001)	19.23 ± 0.36 (*p* = 0.006)

5FU	424.13 ± 3.72 (*p* < 0.001)	39.11 ± 0.73 (*p* = 0.029)	8.45 ± 0.44 (*p* = 0.002)	106.71 ± 1.53 (*p* = 0.003)	14.13 ± 0.35 (*p* = 0.009)

DMH	408.38 ± 1.82 (*p* < 0.001)	32.70 ± 0.88 (*p* = 0.002)	7.26 ± 0.45 (*p* = 0.001)	98.33 ± 2.95 (*p* < 0.001)	10.34 ± 0.29 (*p* < 0.001)

DMH + D1^7 weeks^	458.81 ± 2.42 (*p* < 0.001, ^*∗*^ *p* = 0.002, ^$^ *p* = 0.001)	41.64 ± 0.56 (*p* = 0.008, ^*∗*^ *p* = 0.003)	9.42 ± 0.12 (*p* = 0.042, ^*∗*^ *p* = 0.008, ^$^ *p* < 0.001)	114.23 ± 2.8 (*p* = 0.009, ^*∗*^ *p* = 0.033)	12.30 ± 0.15 (*p* < 0.001, ^*∗*^ *p* = 0.004, ^$^ *p* = 0.017)

DMH + D1^27 weeks^	444.05 ± 3.24 (*p* < 0.001, ^*∗*^ *p* = 0.001)	37.16 ± 0.4 (*p* < 0.001, ^*∗*^ *p* = 0.02, ^$^ *p* = 0.002)	8.82 ± 0.18 (*p* = 0.007, ^*∗*^ *p* = 0.037, ^$^ *p* = 0.027)	104.72 ± 2.39 (*p* < 0.001, ^*∗*^ *p* = 0.041, ^$^ *p* = 0.001)	11.10 ± 0.15 (*p* < 0.001, ^*∗*^ *p* = 0.049, ^$^ *p* < 0.001)

DMH + 5FU	443.82 ± 2.39 (*p* < 0.001, ^*∗*^ *p* = 0.001)	40.21 ± 0.67 (*p* = 0.001, ^*∗*^ *p* = 0.001)	8.16 ± 0.16 (*p* = 0.002, ^*∗*^ *p* = 0.047)	111.32 ± 3.27 (*p* = 0.029, ^*∗*^ *p* = 0.004)	14.2 ± 0.61 (*p* = 0.002, ^*∗*^ *p* = 0.001)

^#^ *p* values indicated in comparison with control; ^*∗*^compared to DMH group; ^$^compared to DMH + 5FU group: for DMH + D1^7  weeks^ and DMH + D1^27  weeks^ groups.

**Table 4:** Parameters of jejunal mucosa for rats treated by D1 and 5FU under DMH-induced carcinogenesis. Mean ± SEM, *n* = 10^#^.

	Mucosa thickness, *μ*m	Enterocytes height, *μ*m	Enterocytes nuclei cross-sectional area, *μ*m^2^	Goblet cells cross-sectional area, *μ*m^2^	Mitotic index, %
Control	636.92 ± 23.15	23.20 ± 1.11	25.68 ± 1.27	112.24 ± 6.48	4.40 ± 0.45

D1^7 weeks^	793.08 ± 53.08 (*p* = 0.002)	22.00 ± 1.24	27.32 ± 1.75	102.60 ± 6.97	5.22 ± 0.43

D1^27 weeks^	720.85 ± 39.5 (*p* = 0.005)	22.13 ± 1.58	19.49 ± 1.16 (*p* = 0.008)	95.63 ± 5.24 (*p* = 0.001)	6.70 ± 0.65 (*p* = 0.032)

5FU	681.01 ± 18.56 (*p* = 0.49)	26.31 ± 1.03 (*p* = 0.001)	22.41 ± 1.62 (*p* = 0.038)	107.11 ± 5.78	5.95 ± 0.59 (*p* = 0.028)

DMH	627.75 ± 20.65	23.20 ± 0.90	22.45 ± 1.6 (*p* = 0.04)	106.24 ± 6.36	5.38 ± 0.96

DMH + D1^7 weeks^	756.73 ± 24.67 (*p* < 0.001, ^*∗*^ *p* < 0.001, ^$^ *p* < 0.001)	25.60 ± 0.94 (*p* = 0.048, ^*∗*^ *p* = 0.044, ^$^ *p* = 0.04)	19.05 ± 1.04 (*p* < 0.001, ^*∗*^ *p* = 0.022)	100.24 ± 6.73 (*p* = 0.047)	5.81 ± 0.4 (*p* = 0.042, ^$^ *p* = 0.008)

DMH + D1^27 weeks^	800.06 ± 41.61 (*p* < 0.001, ^*∗*^ *p* < 0.001, ^$^ *p* < 0.001)	26.14 ± 1.22 (*p* = 0.023, ^*∗*^ *p* = 0.003, ^$^ *p* = 0.013)	21.60 ± 1.29 (*p* = 0.004)	94.42 ± 6.5 (*p* = 0.004, ^*∗*^ *p* = 0.048)	4.53 ± 0.45

DMH + 5FU	636.70 ± 35.05	23.33 ± 1.30	19.09 ± 1.03 (*p* < 0.001, ^*∗*^ *p* = 0.008)	97.40 ± 5.62 (*p* = 0.007)	3.72 ± 0.33 (^*∗*^ *p* = 0.041)

^#^ *p* values indicated in comparison with control; ^*∗*^compared to DMH group; ^$^compared to DMH + 5FU group: for DMH + D1^7  weeks^ and DMH + D1^27  weeks^ groups.

**Table 5:** Liver parameters for rats treated by D1 and 5FU under DMH-induced carcinogenesis. Mean ± SEM, *n* = 10^#^.

	Centrolobular lobulae		Periportal lobulae		Sinusoids diameter, *μ*m
	Hepatocyte cross-sectional area, *μ*m^2^	Hepatocyte nuclei cross-sectional area, *μ*m^2^	Hepatocyte cross-sectional area, *μ*m^2^	Hepatocyte nuclei cross-sectional area, *μ*m^2^	
Control	310.07 ± 3.03	46.23 ± 0.31	282.74 ± 2.58	45.21 ± 0.28	4.37 ± 0.08

D1^7 weeks^	348.45 ± 2.14 (*p* < 0.001)	48.57 ± 0.33	318.47 ± 2.78 (*p* = 0.002)	46.16 ± 0.32	4.81 ± 0.07 (*p* = 0.022)

D1^27 weeks^	339.63 ± 2.47 (*p* = 0.001)	47.93 ± 0.34	309.55 ± 2.66 (*p* = 0.004)	46.1 ± 0.33	4.51 ± 0.09

5FU	333.98 ± 2.51 (*p* = 0.001)	45.32 ± 0.32	317.98 ± 2.84 (*p* = 0.002)	44.25 ± 0.27	4.52 ± 0.07

DMH	366.61 ± 1.50 (*p* < 0.001)	55.59 ± 0.16 (*p* = 0.003)	350.5 ± 1.97 (*p* < 0.001)	51.02 ± 0.34 (*p* = 0.003)	5.12 ± 0.04 (*p* = 0.019)

DMH + D1^7 weeks^	364.32 ± 1.81 (*p* < 0.001)	55.49 ± 0.15 (*p* = 0.002)	358.07 ± 1.77 (*p* < 0.001)	52.04 ± 0.29 (*p* = 0.001)	5.31 ± 0.04 (*p* = 0.001)

DMH + D1^27 weeks^	370.58 ± 1.30 (*p* < 0.001, ^$^ *p* = 0.002)	54.06 ± 0.22 (*p* = 0.002, ^$^ *p* = 0.039)	351.86 ± 2.11 (*p* < 0.001)	50.89 ± 0.30 (*p* = 0.008)	4.95 ± 0.06

DMH + 5FU	355.97 ± 2.00 (*p* < 0.001, ^*∗*^ *p* = 0.015)	55.82 ± 0.14 (*p* = 0.001)	349.06 ± 2.19 (*p* < 0.001)	52.19 ± 0.28 (*p* = 0.001)	5.04 ± 0.04 (*p* = 0.004)

^#^ *p* values indicated in comparison with control; ^*∗*^compared to DMH group; ^$^compared to DMH + 5FU group: for DMH + D1^7  weeks^ and DMH + D1^27  weeks^ groups.

**Table 6:** Blood serum parameters for rats treated by D1 and 5FU under DMH-induced carcinogenesis. Mean ± SEM, *n* = 10.

	ALT, nmol pyruvate/mg protein per min	AST, nmol pyruvate/mg protein per min	ALP, nmol 4-nitrophenol/mg protein per min	LDH, nmol pyruvate/mg protein per min
Control	0.43 ± 0.04	0.57 ± 0.04	7.3 ± 0.27	14.6 ± 2.7
D1^7 weeks^	0.32 ± 0.04^*∗*^	0.57 ± 0.05	7.57 ± 0.22	13.5 ± 2.16
D1^27 weeks^	0.34 ± 0.03^*∗*^	0.59 ± 0.07	7.3 ± 0.27	14.04 ± 2.7
5FU	0.39 ± 0.03	0.44 ± 0.07^*∗*^	7.01 ± 0.25	13.9 ± 2.9
DMH	0.52 ± 0.03^*∗*^	0.43 ± 0.04^*∗*^	10.38 ± 0.32^*∗*^	16.2 ± 3.24
DMH + D1^7 weeks^	0.52 ± 0.02^*∗*^	0.54 ± 0.04^#^	7.35 ± 0.27^#^	14.31 ± 2.7
DMH + D1^27 weeks^	0.52 ± 0.04^*∗*^	0.53 ± 0.05^#^	7.14 ± 0.32^#^	13.5 ± 2.43
DMH + 5FU	0.53 ± 0.04^*∗*^	0.53 ± 0.04^#^	7.42 ± 0.31^#^	15.3 ± 2.57

^#^ *p* values indicated in comparison with control; ^*∗*^compared to DMH group.

## Abbreviations

D1: 5-Amyno-4-(1,3-benzothyazol-2-yn)-1-(3-methoxyphenyl)-1,2-dihydro-3H-pyrrol-3-one
DMH: 1,2-Dimethylhydrazine
AOM: Azoxymethane
MI: Mitotic index
ALT: Alanine aminotransferase
AST: Aspartate aminotransferase
ALP: Alkaline phosphatase
LDH: Lactate dehydrohenase
8-oxoG: 7,8-Dihydro-8-oxoguanine
Yes: Yamaguchi sarcoma viral oncogene homolog 1
Src(h): Rous sarcoma oncogene cellular homolog
ZAP-70: Zeta-chain-associated protein kinase 70
Syk(h): Spleen tyrosine kinase
PDK1: 3-Phosphoinositide-dependent kinase 1
TGF*β*: Tumor growth factor *β*
PI3K: Phosphatidyl-inositol-3 kinase
COX-2: Cyclooxygenase-2
TNF*α*: Tumor necrosis factor *α*
PGE2: Prostaglandin E2
EGFR: Endothelial growth factor receptor
IGF1R: Insulin-like growth factor-1 receptor
VEGFR: Vasoendothelial growth factor receptor
TKI: Tyrosine kinase inhibitor
ABC transporter: ATP-binding cassette transporter
OATP: Organic anion transporting polypeptide
PPAR-delta: Peroxisome proliferator-activated receptor-delta
TLR-4: Toll-like receptor 4
JAK/STAT: Janus kinase/signal transducer and activator of transcription pathways
PDK1: 3-Phosphoinositide-dependent protein kinase 1
dTMP: Deoxythymidine monophosphate
FdUTP: Fluorodeoxyuridine triphosphate
ROS: Reactive oxygen species.
